# High-resolution portable bluetooth module for ECG and EMG acquisition

**DOI:** 10.1016/j.csbj.2025.04.020

**Published:** 2025-04-15

**Authors:** Luiz E. Luiz, Salviano Soares, Antonio Valente, João Barroso, Paulo Leitão, João P. Teixeira

**Affiliations:** a Research Centre in Digitalization and Intelligent Robotics (CeDRI), Laboratório Associado para a Sustentabilidade e Tecnologia em Regiões de Montanha (SusTEC), Instituto Politécnico de Bragança, Bragança, 5300-253, Portugal; b Engineering Department, School of Sciences and Technology, University of Trás-os-Montes and Alto Douro (UTAD), Quinta de Prados, 5000-801, Vila Real, Portugal; c Institute of Electronics and Informatics Engineering of Aveiro (IEETA), University of Aveiro, Aveiro, 3810-193, Portugal; d Intelligent Systems Associate Laboratory (LASI), University of Aveiro, Aveiro, 3810-193, Portugal; e INESC TEC—INESC Technology and Science, Porto, 4200-465, Portugal; f INESC TEC-Instituto de Engenharia de Sistemas e Computadores, Tecnologia e Ciência, Polo da UTAD, 5000-801, Vila Real, Portugal

**Keywords:** Electrocardiogram, Electromyogram, Printed circuit board, Acquisition integrity, Telemedicine, Wearable, Human-centred

## Abstract

Problem: Portable ECG/sEMG acquisition systems for telemedicine often lack application flexibility (e.g., limited configurability, signal validation) and efficient wireless data handling. Methodology: A modular biosignal acquisition system with up to 8 channels, 24-bit resolution and configurable sampling (1–4 kHz) is proposed, featuring per-channel gain/source adjustments, internal MUX-based reference drive, and visual electrode integrity monitoring; Bluetooth® transmits data via a bit-wise packet structure (83.92% smaller than JSON, 7.28 times faster decoding with linear complexity based on input size). Results: maximum 6.7 μV${}_{\text{rms}}$ input-referred noise; harmonic signal correlations >99.99%, worst-case THD of -53.03 dBc, and pulse wave correlation >99.68% in frequency-domain with maximum NMSE% of 6e-6%; and 22.3-hour operation (3.3 Ah battery @ 150 mA). Conclusion: The system enables high-fidelity, power-efficient acquisition with validated signal integrity and adaptable multi-channel acquisition, addressing gaps in portable biosensing.

## Highlights

• Parallel acquisition of ECG and sEMG with portable module.
• Up-to 8 channels of 24-bit ΣΔ acquisition and adjustable sampling frequency.
• Acquisition transmission and configuration through Bluetooth® communication.
• Packages 83.92% smaller with linear decode complexity, 7.28x faster than JSON.
• Maximum input-referred noise of 6.7 μV_rms_, THD < -56 dBc, and signal correlation >99.99%.

## Graphical abstract

**Figure:**
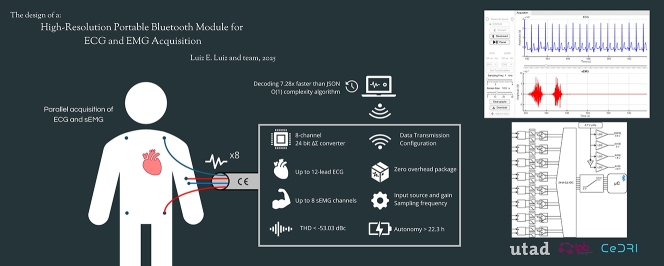

## 1 Introduction

Improvement in the analysis of humans' daily routine through biosignal processing provides big data for earlier detection of diseases, increases the effectiveness of treatment [1], and optimises athletes' muscular gain and recovery [2], allowing physicians a better follow-up in cardiovascular and muscular rehabilitation.

Multichannel biosignals' real-time acquisition and analysis have become increasingly important in various biomedical applications, including clinical monitoring, diagnostics and scientific research [3]. The need for robust and efficient systems that can acquire, process and transmit these signals in real-time is fundamental to ensuring the accuracy and effectiveness of modern medical applications [4].

Biosignals, such as the electrocardiogram (ECG) and electromyogram (EMG), contain valuable information about an individual's physiological state. However, these signals' complexity and unpredictability nature require advanced tools for their acquisition and analysis. Extracting relevant characteristics from these signals is essential for accurate interpretation and clinical decision-making, easing complex analysis.

The electrocardiogram (ECG) visually represents the heart's functioning through electrical measurements. The resulting maximum peak voltage reaches up to 5 mV, changing depending on conditions such as sex and age [5]. The ECG frequency band depends on the intended objective. The most comprehensive typically range from 0.5 Hz to 150 Hz [6]. The ECG medical-grade acquisition using 10 electrodes can be uncomfortable. Therefore, alternative configurations with only three electrodes are used [7], [8], with the electrodes placed equidistant from the heart, known as the Einthoven triangle [7], [9].

The electromyogram (EMG) represents muscle contraction through electrical measurement [10]. The surface electromyogram (sEMG) is the non-intrusive way of acquiring EMG signals, typically using one pair of electrodes per muscle plus a reference [11], [12]. The maximum amplitude varies with the contraction intensity, muscle, strength, sex, and age [13]. The frequency bandwidth stays within 10 to 500 Hz [11], [14], [10]. Its applications include medical diagnosis, muscle rehabilitation and the control of electric prostheses [11].

Some developments conceive this objective of a portable analysis of biosignals using synthetical simulator-generated signals to test its results. In [15], the acquisition module was developed, but the signal came from a potential simulator. Other developments depend on pre-recorded data, such as [16] developing an interface for interpreting ECG from pre-existing datasets, where certain frequency bands may have already been processed, restricting some studies beyond the standard frequency bands. Similarly, there are EMG-focused systems that start from a pre-existing database, as in [11] or [17], use pre-existing acquisition systems, as in [14], or without a focus on processing the signal parameters, as in [10].

Some studies include hardware and interfaces but focus on a single biosignal. In [18], a 16-bit ECG acquisition is sent via USB to a mobile device, which then transmits it to a server where the signals are presented graphically. In [19], the development was aimed at education, using frequency bands that prioritise the clear visual recognition of ECG points instead of a complete signal's frequency spectrum.

There are also developments focused on specific applications. In [20], an sEMG acquisition was created as feedback for an electrical stimulation system for rehabilitation. The data is sent to a mobile phone using BLE (Bluetooth® low-energy), which calculates electrical stimulation parameters based on the patient, pathology, and sEMG reading.

The work [21] presented a wearable acquisition system for signals, including sEMG and ECG, sent to a mobile phone using BLE. This system creates a platform to predict fall occurrences and electrical stimulation routines to prevent falls.

Using ECG and sEMG as feedback in portable rehabilitation systems is solid in the state-of-the-art to improve patient safety and personalised treatment [20], [21]. However, multipurpose systems that seek ECG and sEMG acquisition trade off their signal-specific analogue conditioning blocks to make the system versatile [22]. These systems require the user to select the wanted signal before the acquisition, as in [23].

From the presented systems, it is clear that there is a preference for BLE protocol in wireless communication to reduce power consumption. However, BLE is preferable in applications where the data is sparse in time, meaning that for this application, its reduced consumption is deprecated. At the same time, BLE also has a reduced data transmission volume, constraining larger sampling frequencies or data resolution.

Another topic that requires improvement is the resolution of signal conversion, where common wireless commercial devices support a maximum of 10-bit conversion with only 4 channels [24], 14-bit sampled at 300 kHz for intermittent 6 leads [25], or 24-bit with only 1 channel [26].

Although wireless signal transmission brings the added value of not connecting the user to a third-party device, wireless protocols are limited regarding the size of data packets. This leads developers to choose lower-resolution ADC, smaller sampling frequency or fewer channels to comply with this size constraint. However, acquisition modules developed for electrophysiology signal acquisition should have an input-referred noise of less than 5 μV${}_{\text{rms}}$ [27], not achievable with less than 20-bit resolution ADC with a symmetrical range of 2.4 V without analogue gain (a least significant bit of 4.58 μV${}_{\text{rms}}$). Therefore, if a smaller number of analogue conditioning parts is defined as objective, the ADC should have a resolution higher than 20-bit. This also increases the module flexibility, as it becomes less dependent on immutable hardware Regarding flexibility within the frequency band of conditioning, commercial devices such as BioPac MP35 and BITalino (r)evolution have predefined conditioning circuits that limit studies that explore flexible bands [28]. Another lack of flexibility is noticed with possible sampling frequencies, kept at a maximum of 1 kHz [24] (minimum sEMG sampling frequency [11]) to fit in wireless bandwidth, constraining other signal analyses [25]. Commercial devices also limit the cables that the system is compatible with instead of ensuring compatibility with medical-grade certified cables.

These constraints limit end-users, whether through immutable hardware/software configurations or delayed data transmission.

This work, focusing on adaptability to user workflows and environments as a human-centred design principle, introduces a modular, wirelessly accessible acquisition platform designed to prioritize a flexible user operation. By integrating configurable multi-channel architecture (1-8 channels, 24-bit resolution), intuitive gain configuration, signal source adjustments, and electrode integrity monitoring, the system seeks to allow clinicians and researchers to flexibly and reliably acquire biological signals. Simultaneously, its optimized bit-wise Bluetooth® packet structure aims to overcome traditional bandwidth limitations.

The work is divided into this introduction, followed by a section focused on the module design, explaining the hardware developed, the firmware algorithm, and the interface to control the module and receive the data. Then, the results are shown as validation of the acquired data, followed by the conclusion.

## 2 Design

The proposed system encompasses the acquisition module (i.e., hardware), microcontroller algorithm (i.e., firmware) and the user interface to control the acquisition and visualize the results. The design section is equally divided to expose the methods and the resulting development.

### 2.1 Hardware description

**Fig. 1:**
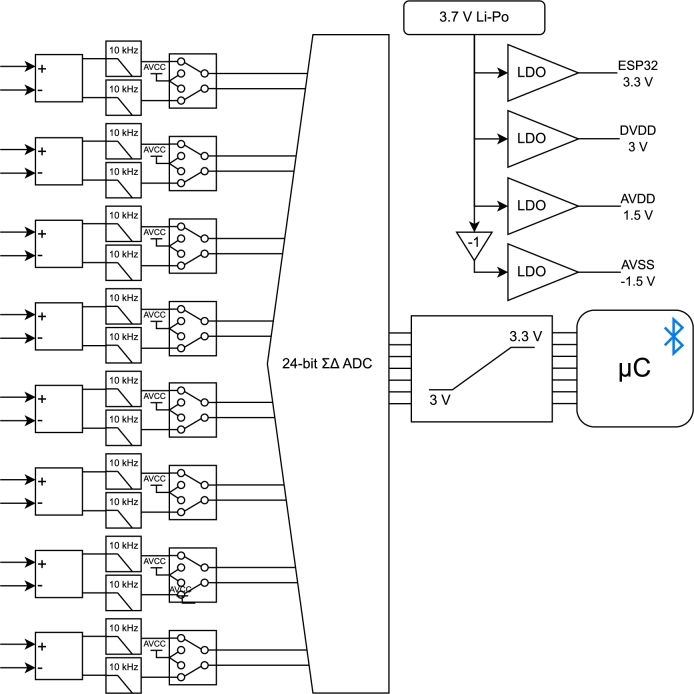
Hardware block structure.

Oriented by the requirements for the PCB (printed circuit board), a hardware block design was developed as a reference for the development, as shown in Fig. 1.

From the PCB external connection, medical-grade compatible cables are used to maintain flexibility, with a snap-button connector for the electrode and D-sub for the PCB. Furthermore, an audio jack input is also included as one of the channels for an easily ready-to-use cable with the same snap-button connector for the electrodes but with a 3-pole audio output to connect to the PCB. This ambiguity facilitates user interaction when using the module, depending on the number of required channels, adapting the module to the real and different needs of users as a human-centred design principle. Commercial devices lack this flexibility, making the module require a company-specific cable with less certification than medical grade (e.g., BioPac MP35 and BITalino (r)evolution [24]).

Within the PCB, the initial objective is to have multiple channel inputs, a high-resolution analogue-to-digital converter (ADC) and easily configurable parameters. Based on the state-of-the-art, the ADS129x integrated circuit (IC) family was selected as the analogue front end of the acquisition module. This IC family has a 24-bit resolution ADC, delta-sigma modulation, highly configurable with programmable gain (1 to 12), configurable sampling frequency, and internal multiplexers for Right-Leg-Drive (RLD) configuration and Wilson Central Terminal (WCT). Multiplexing the input to read different PCB-internal features, such as input noise, power supply, and temperature, is also possible.

The supply was projected to focus on portability, requiring one 18650 3350 mAh Li-Ion battery with a charge pump voltage inverter for the negative voltage and voltage regulator for the different ADS129x supply requirements. Namely, in this application, the ADS129x requires a symmetrical analogue supply (+-2.5 V) and a digital supply (+3 V), and the microcontroller requires another digital supply (+3.3 V).

A linear voltage regulator was used for every positive voltage supply. The negative value was achieved by inverting the battery voltage and then connecting it to a negative voltage regulator. This order is required as the inverter is not regulated, but the ADS129x requires a highly stable, noise-free supply to achieve good results.

All the inputs were connected to an antialiasing RC (resistor-capacitor) low-pass filter with a cut-off frequency of 10 kΩ to reduce the signal bandwidth. The path is then interrupted by channel-independent mechanical switches that turn the inputs manually on and off if required. If the channel is not used, the switch can be turned off, and the input's positive and negative sides will be short-circuited to the analogue positive supply, as recommended by the IC manufacturer. This change does not affect the ADC channels, which remain active if required.

The chosen microcontroller was the ESP32, which, based on the literature, provided embedded wireless communication circuitry with flexibility regarding communication protocols, with high operating clock frequency, reaching 240 MHz with two independent cores. For the PCB, its ESP32-DevKitC-32D model was used to provide easy access to external pins as a prototype design.

As the ADS129x digital supply is +3 V and, therefore, its SPI protocol drives this 3 V as a digital high, a logic level translator was used to ease digital communication between the ADS129x and the ESP32, since its digital high is 3.3 V. Although communication could result in success based on the susceptibility to lower voltages, this reduces pressure and allows viable higher communication frequency [29].

The PCB was designed with low-tolerance surface-mount components to increase reliability and linearity and reduce weight and size.

### 2.2 Firmware description

**Fig. 2:**
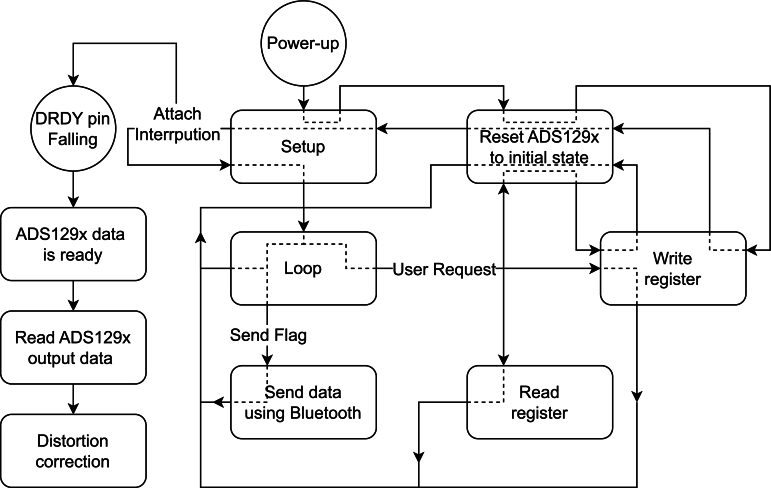
Track of the developed microcontroller firmware as a block diagram.

The firmware follows the algorithm summarised in Fig. 2.

After the power-up, the algorithm starts the setup function. The setup function initializes the Bluetooth® device, then initiates the ADS129x reset routine configuring the IC registers. Finally, it creates the interruption that represents the data-ready function.

The data ready function is an interruption attached to the DRDY pin from the ADS129x. The “read ADS129x output” function is activated when a falling edge is detected. This function concatenates every sample in the acquisition buffer and increases the sample counter. Suppose the sample counter reaches the number of messages per package (40 messages, each comprising one time and one sample of each requested channel, e.g., an ECG and a sEMG sample). In that case, this function will clear the counter, copy the information from the acquisition buffer to the transmission buffer and activate the sending flag.

**Fig. 3:**
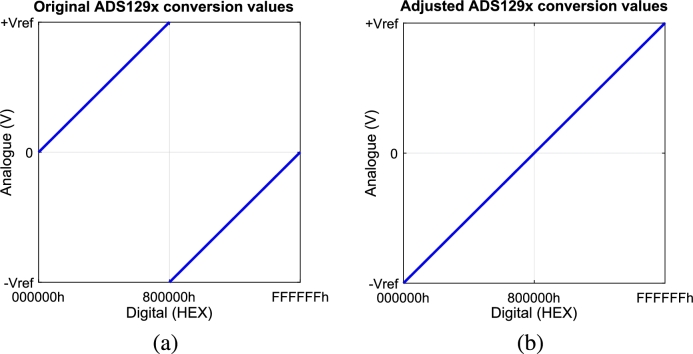
Representation of correlation between analogue and digital values. (a) represents the original translation in the ADS129x's output. (b) represents the resulting translation after the distortion correction function is applied.

The read ADS129x output data function is activated inside the data ready function. It reads the ADS129x SPI output, which comprises 24 bits per available channel (i.e., 4 channels in ADS1294, 6 in ADS1296(R) and 8 in ADS1298(R)), plus 24 status bits that have information on the ADS129x GPIOs and input individual connection (i.e., contains information regarding connection condition of every positive and negative electrode). This function also initiates a tertiary function to correct polarity inversion on the binary to voltage scales. As shown in Fig. 3, the negative and positive analogue values are inverted in the digital scale; the positive analogue values vary between 0x000000 and 0x7FFFFF, and the negative analogue values are placed from 0x800000 until 0xFFFFFF. The tertiary function adjusts this inversion to maintain the values linearly. It is made by subtracting 0x800000 from values higher than 0x7FFFFF and adding 0x800000 to values lower than 0x7FFFFF

While the algorithm waits for interruption, it enters the main loop. From this point, the algorithm has different branches depending on the situation.

The send data function is activated when the send flag is on during the loop function. It transmits the transmission buffer through the Bluetooth® channel.

The read and write registers are activated inside different secondary functions or through user requests. They are programmed to aid in the ADS129x register configuration while maintaining the SPI-specific timing requirements.

The ADS129x reset function can also be activated from inside the loop when the GUI resets or initiates, forcing the ADS129x to initiate in a known state. All the configuration is done register-wise.

The ADS129x initial state is defined as a known base when the system initiates, or the module receives a reset command. In CONFIG1 (0x01), the output data rate (i.e., sampling rate) is set to 1 kSPS (0x85). In CONFIG3 (0x03), the RLD buffer is turned on for RLD usage, and the reference voltage is set to 2.4 V (0xDC). In CONFIG4 (0x17), the comparators used for electrode connection integrity are turned on (0x02). In LOFF (0x04), the electrode connection integrity drive is defined as an AC source signal with a 6 nA current and a threshold of 95% (0x03). In RLD_SENSP (0x0D), the positive source of the RLD is defined as the channel 1 positive input (0x01). Similarly, in RLD_SENSN (0x0E), the negative source of the RLD is defined as the channel 1 negative input (0x01). Then, all the channels (0x05 to 0x0C) are set to have normal electrode input with a gain of 12x (0x60). Finally, in WCT1 (0x18), the first (of three) WCT comparator is turned on and connected to the negative side of input 1 (0x09). In WCT2 (0x19), the second comparator is turned on and connected to the negative side of input 2, and the third comparator is turned on and connected to the positive side of input 1 (0xD8). The final register map, including the registers kept in their reset values, is shown in Table 1.

**Table 1:** Register map configured as the firmware's initial state.

Address	Register	Bit							
		7	6	5	4	3	2	1	0
0x00	ID^1^	x	x	x	x	x	x	x	x
0x01	CONFIG1	1	0	0	0	0	1	0	1
0x02	CONFIG2	0	0	0	0	0	0	0	0
0x03	CONFIG3	1	1	0	1	1	1	0	0
0x04	LOFF	0	0	0	0	0	0	1	1
0x05 to 0x0C	CHxSET	0	1	1	0	0	0	0	0
0x0D	RLD_SENSP	0	0	0	0	0	0	0	1
0x0E	RLD_SENSN	0	0	0	0	0	0	0	1
0x0F	LOFF_SENSP	0	0	0	0	0	0	0	0
0x10	LOFF_SENSN	0	0	0	0	0	0	0	0
0x11	LOFF_FLIP	0	0	0	0	0	0	0	0
0x12	LOFF_STATP	0	0	0	0	0	0	0	0
0x13	LOFF_STATN	0	0	0	0	0	0	0	0
0x14	GPIO	0	0	0	0	1	1	1	1
0x15	PACE	0	0	0	0	0	0	0	0
0x16	RESP	0	0	0	0	0	0	0	0
0x17	CONFIG4	0	0	0	0	0	0	1	0
0x18	WCT1	0	0	0	0	1	0	0	1
0x19	WCT2	1	1	1	1	1	0	0	0

1 The ID register is a read-only register that stores information regarding the device-specific characteristics within the ADS129x family. Considering that the IC used in this development was the ADS1296, a reading command in the ID register would return the binary word 10011001.

From within the loop function, the data received from the Bluetooth® channel is also read. Every package is a byte representing the user's configuration in the GUI. Converting the byte to decimal: 1, 2 and 4 represent sampling frequency changes, respectively 1, 2 and 4 kSPS; 10 plays the acquisition/transmission and 11 pauses it; 12 enters in the system test routine and transmits to the interface all RLD, WCT and input connection state; 14 ends the system test routine; 20 to 28 defines the first transmitted signal source, the possible options are the status word or any of the 8 channels (considering the ADS1298); 30 to 38 defines the second transmitted signal source; 201 is a register-write command; and 255 is an ADS129x reset command.

Considering that the interface developed is still compatible with only two simultaneous graphs, the channels that are not requested are turned off. Similarly, when manually acquiring the data (with no interface), the user can have between 1 and 8 channel readings, where the channels that are not requested are equally turned off to reduce power consumption.

Furthermore, when the system test mode is activated, the acquisition is already paused; the system, therefore, sends the GUI data regarding each positive and negative input connection status and which ADS129x it is (i.e., ADS1294, ADS1296 or ADS1298 and if it has respiration functionality, represented by the suffix R (ADS1298R)) for the debugging process.

### 2.3 Controller interface

A GUI was developed inside the MATLAB R2021a App Designer, resulting in a stand-alone application that does not require any MATLAB subscription to use the application.

Since the objective was to control the module interactively, this initial interface is compatible with only two parallel graph plots.

It was developed using an object-oriented algorithm in an event-driven approach, attaching specific functions to specific GUI events and a respective priority between functions. Therefore, the GUI algorithm is not linear, and it is inherently based on how the user intends to use the software.

**Fig. 4:**
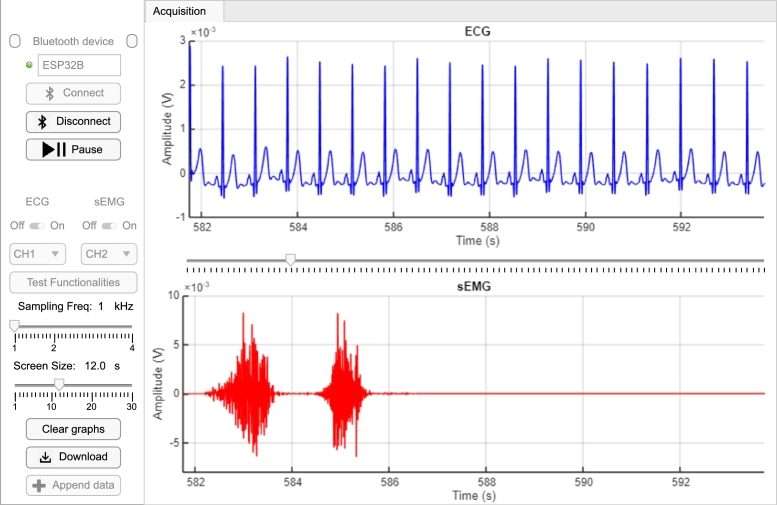
GUI in acquisition tab with sample signal plotted. The controls are positioned on the left, with: Bluetooth® options to connect, disconnect, pause or resume acquisition; two selectors for the channel that will source the graphs; a sampling frequency slider for 1, 2 or 4 kHz; a graph sliding window size; and buttons to clear or download the acquired data. On the right, the two graphs show the acquired signal in real-time. Between the graphs, when the acquisition is paused, the user can scroll the window back to visualize the previous.

Using the module presented in this work, a sample signal was acquired to act as an example and to be shown in the interface figures. The sample signal was acquired using the hardware module and recorded through the interface. The CS5 lead was used for the ECG, and the reference electrode served as RLD based on the other two ECG potential electrodes. For the sEMG, the two potential electrodes were connected 3 cm apart over the biceps, with the reference electrode connected to the elbow, with two medium-intensity consecutive contractions. The signal had a sampling frequency of 1 kHz. The resulting signal is shown in Fig. 4.

The interface allows the user to connect, pause and disconnect from the module Bluetooth® channel. The user can also download or clear the data acquired. The user can also select which channel should appear in each of the graphs presented in the interface.

**Fig. 5:**
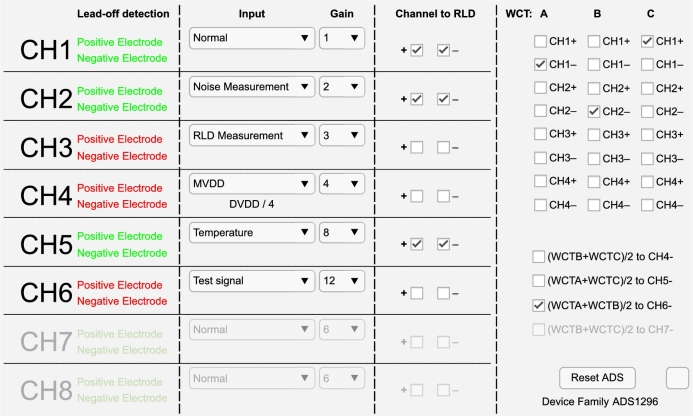
The test functionalities window in connected state and acquisition paused.

Furthermore, when the user pauses the acquisition without disconnecting, the “Test Functionalities” button is enabled, allowing the user to enter a new screen where the ADS129x can be tested and/or visually edit the registers' configuration. This new window is shown in Fig. 5. When the user enters this configuration state, the previous screen gets temporarily disabled.

While in the test functionalities window, Fig. 5, the user can see which of the available channels have its positive and/or negative electrode correctly connected to the patient body, green name representing connection and red representing disconnection (i.e., in Fig. 5, both positive and negative electrodes are connected in channels 1, 2 and 5, and not connected in channels 3, 4 and 6). Furthermore, since the ADS129x in the connected device is the ADS1296 (information gathered by the ID register and shown in the GUI's southeast corner), channels 7 and 8 are disabled in Fig. 5.

The user can change the channel input source between Normal Electrode (as in CH1), Channel Noise Measurement (as in CH2), RLD Measurement (as in CH3), Power Supply Measurement (as in CH4), Temperature (as in CH5), Internal Test Signal (as in CH6), and drive the RLD through the channel's positive or negative electrode. It can also change the channel-specific gain between the IC's PGA (programmable gain) options (i.e., 1, 2, 3, 4, 6, 8 or 12 V/V).

As for the RLD information, the user can choose which positive and/or negative is required to calculate the RLD signal. Any available channels, positive and negative electrodes, can be routed to the RLD driver.

For the Wilson Central Terminal, the user can choose one electrode from channels 1 to 4 for each of the three WCT comparators and can route the mean value of pairs of the WCT comparators to channels 4 to 7 (if available) negative electrode. This allows the user to measure augmented ECG leads.

Finally, the user can reset the ADS129x to the initial configuration shown in Table 1.

When the test functionalities window is opened, the acquisition module sends all the actual IC registers to the GUI to adjust the interface to the real state. The lead-off detection status is updated every 0.5 s. After all the changes have been made, when the user closes the window, all the new configuration is sent to the hardware module, which will then write the registers with the new values.

Each register's new data is sent to the module as a binary 8-bit word. Inside the microcontroller firmware, these packages are handled inside the loop function, as mentioned in Section 2.2. For every word, one write register command is sent to the ADS129x. Likewise, all the required registers are read through a read register command at the start of the test functionalities. As for the lead-off continuous refresh, to avoid delivering two read register commands every refresh period of 0.5 s (one for positive electrodes and another for negative electrodes), this information is gathered from the status word present on the ADS129x's normal output data stream, mentioned in the Section 2.2.

## 3 Results

**Fig. 6:**
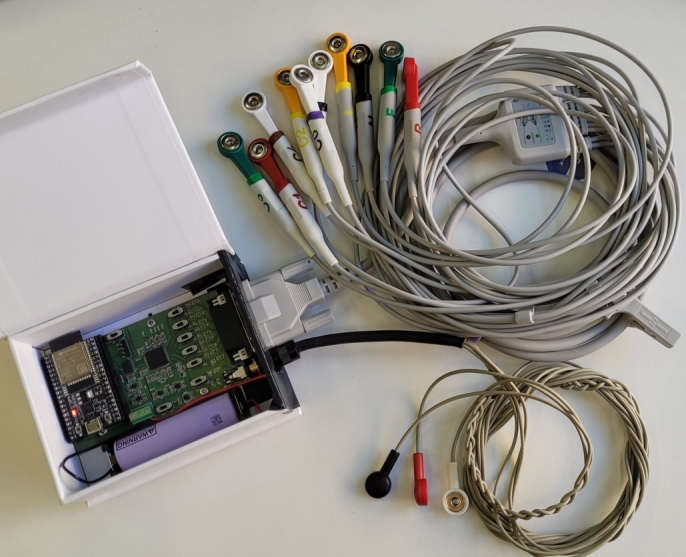
Assembled module with 10-electrode medical grade cable and 3-electrode to audio jack cable.

To validate the development, one prototype was assembled and tested. The ADS1296 was used in this prototype. The assembled module is shown in Fig. 6 with both electrode cables (i.e., the 10-electrode medical grade cable and the 3-electrode to audio jack cable).

### 3.1 Battery autonomy evaluation

When the module is turned on, it automatically enters Bluetooth® pairing mode. In this state and with all ADC channels turned on, the system draws a mean of 72 mA from the battery. If a receiver device is connected, the system enters the acquisition mode, which draws a mean of 150 mA. In both states, 9 mA was used to power the ADS1296, the logic level translator IC, and all LDO. The ESP32 development board used the remaining current.

The current draw by the PCB changes regarding the number of ADC channels activated. Excluding the current used by the microcontroller, the maximum remaining current with all 6 channels activated is 9 mA, reducing approximately 0.8 mA for each deactivated channel, reaching a minimum of 4.2 mA when no channel is on.

Two tests were made with two different 18650 3350 mAh Li-Ion batteries fully charged, connecting the system to a receiver and sending data from all 6 channels until turning off. On the first, the module acquired and sent data during 22 hours and 20 minutes and on the second, during 22 hours and 43 minutes.

### 3.2 Parallel acquisition

**Fig. 7:**
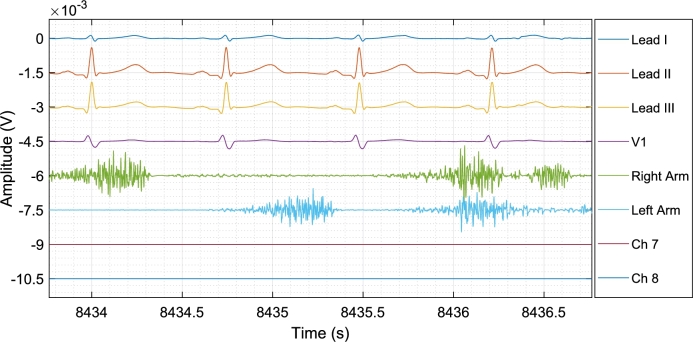
Acquisition of the 8 simultaneous signals from the module. Channels 1 to 6 represent the ECG leads I, II, III, and V1, and sEMG from right and left flexor carpi radialis. Channels 7 and 8 are not accessible in the ADS1296 used, resulting in a constant 0 V. A factor of 1.5 mV shifts the signals to improve visibility.

To test the system's maximum parallel acquisition (i.e., 8 channels), an acquisition was made outside of the controller interface. Since the ADS1296 was used in the prototype, the readings of channels 7 and 8 were constant at zero. The remaining channels were connected respectively for: ECG lead I, II, III and V1, and sEMG of right and left flexor carpi radialis. The signals were offset by a factor of 1.5 mV and plotted together. This resulted in Fig. 7.

### 3.3 Data formatting comparison

The proposed approach concatenates the raw binary data in periodic sections in a constant stream of information. Therefore, it requires knowledge of how the information is being arranged, synchronism to when the packages start and end, and the package's expected size. Hence, the information is not wrongly read.

The transmitted packets are divided into messages; each message is subdivided into individual samples (one for time plus one for each channel being read); and the samples are divided into bytes (4 bytes for time and 3 bytes for each signal sample)

When using the controller interface, acquiring two parallel signals, the firmware formats the data in periods of 10 bytes, embracing one sample of time, ECG and sEMG in an LSB-first format with 4, 3 and 3 bytes, respectively. To build a package, 40 samples are concatenated in a buffer with a resulting size of 400 bytes that will be sent on the data stream.

To maintain data integrity, the receiver is forced to read the stream in groups of 400 bytes. The Bluetooth® cache is always erased when connected to avoid residual data after the previous disconnection. This same approach must be applied if the transmission is paused and resumed.

The signal integrity was validated in 3 tests of 30 minutes, acquiring signals with a sampling frequency of 1 kHz. During this test, the module was between 1 and 1.5 meters from the receiver. Further distance tests were not evaluated since standard Bluetooth protocols were implemented and real-scenario obstacles are not predictable. The validation was made by checking the time samples received by the module. Since all the timestamps were spaced in 1 ms, it is concluded that no data was corrupted in the transmission.

The communication relies on classic Bluetooth® automatic Cyclic Redundancy Check (CRC). If a packet does not match the CRC at the receiver, it is automatically discarded, and a new sample is requested from the sender. If this 40-sample packet fails to be sent multiple times and the sending vector is overwritten at the sender, the receiver will miss the complete packet (e.g., the complete 40 samples); therefore, the sync will continue.

To validate the efficiency of the proposed Bluetooth® transmission in a bit-wise format, the method is compared against JSON format based on its established use in the literature.

In the bit-wise approach, the package size in the microcontroller is precisely 400 bytes since it is the raw data. However, in the JSON format, for a package with 40 samples of time, ECG and sEMG, testing on 5400000 packages, the resulting size varied between 606 and 862 bytes with a mean of 735.69 bytes. This results in a mean overhead of 335.69 bytes.

On the receiver side, three different algorithms were tested. Two to decode the bit-wise (one based on for loops and the other based on vector reshaping operation) and the third as a standard JSON decode algorithm.

The ‘algorithm 1’ iterates on a matrix of 400xN, where ‘N’ represents the number of messages to read per iteration, casting each sample from binary to 32-bit integers (native size on MATLAB).

**Fig. 8:**
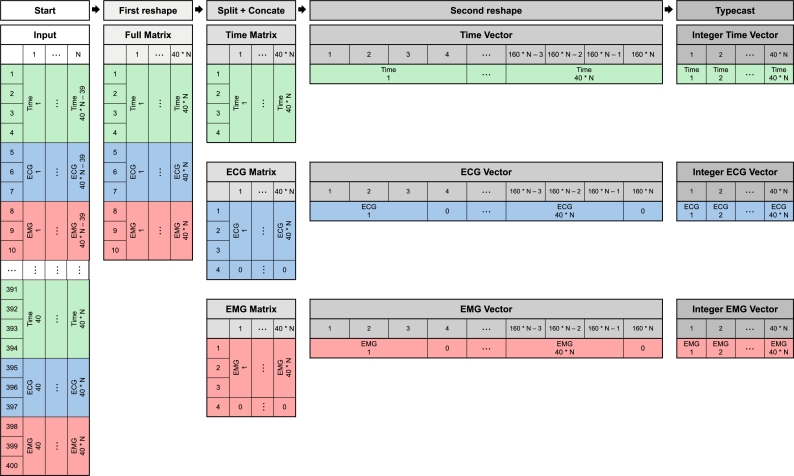
Visual Representation of the data reshaping performed by ‘algorithm 2’.

The ‘algorithm 2’ process is shown in Fig. 8. It takes the 400xN matrix and reshapes it to a 10x40*N, where the 4 first lines are data from the time, the middle 3 from the ECG and the last three from the sEMG. Afterwards, the matrix is divided into three matrices containing only the respective lines (i.e., 4x40*N, 3x40*N, and 3x40*N). To make the three matrices compatible with 32-bit (4 bytes) integer casting, the ECG and sEMG matrices are concatenated with one line of zeros in its least significant byte position. These final matrices are reshaped into three vectors and then cast to 32-bit integers. If a different number of channels were being acquired, the number of input lines would equally increase, reaching a maximum of 1120 bytes per packet when acquiring 8 channels (40 messages containing 4+3*8 bytes).

The ‘algorithm 3’ scans the transmitted data string to find the delimiter, creating a struct with time, ECG and sEMG samples. This approach has increased robustness compared to the bit-wise, as the receiver has a specific delimiter to base the decoding, knowing every message beginning and ending, thus making the package's size irrelevant. Since the JSON approach cannot stack multiple messages, ‘N’ was kept as 1, i.e., the algorithm is tested reading only one message each iteration. In cases where the reading of multiple messages per iteration is tested (N > 1), the algorithm is activated inside a loop to activate it ‘N’ times.

The time required by all three algorithms to decode 1 hour of the signal (90,000 messages) was tested, considering 40 samples per message and 1, 2, 5, or 10 messages per iteration, achieving the result shown in Table 2. Besides the direct time required to decode each iteration, it is also helpful to know how much from the interval between iterations is used to decode the message. Based on this variable, it is possible to estimate the remaining time available to process the signal (e.g., filter, feature extraction).

**Table 2:** Time required per iteration to decode 90,000 data messages with the three different algorithms considering 40 samples per message and ‘N’ messages per iteration.

N	Algorithm	Required Time (μs)			Time usage (%)
		Mean	Min	Max	
1	1	3587.53	307.30	13240.50	3.59%
	2	187.51	14.80	1543.40	0.19%
	3	211.11	45.40	842.60	0.21%

2	1	8048.35	565.50	41491.50	4.02%
	2	231.94	15.70	1000.40	0.12%
	3	467.99	78.60	2055.40	0.23%

5	1	20160.77	1387.40	62911.50	4.03%
	2	235.42	27.10	654.20	0.05%
	3	959.20	202.30	2960.30	0.19%

10	1	35305.62	2754.20	76631.50	3.53%
	2	222.90	36.50	628.80	0.02%
	3	1469.93	398.50	3542.60	0.15%

The case with only one message per iteration (N = 1) was the most straightforward to all three algorithms, allowing a direct comparison of their decoding efficiency. The ‘algorithm 2’ was the most efficient, being, on average, 21.07 times faster than the ‘algorithm 1’ and 1.38 times faster than the ‘algorithm 3’. The ‘algorithm 2’ also achieved the shortest recorded time in a specific iteration. When comparing each algorithm's highest required time, the ‘algorithm 3’ achieves the best result, with the ‘algorithm 2’ in a second. As for the time usage, ‘algorithm 2’ and ‘algorithm 3’ were close at 0.19% and 0.21%, with the ‘algorithm 1’ algorithm being considerably less efficient.

However, in the case studies where it is considered that the user decreased the data refresh rate on the receiver, i.e., increased the number of messages to read between iterations (N > 1), the ‘algorithm 2’ algorithm stood out even more. As the number of messages per iteration grew, the efficiency of the ‘algorithm 2’ algorithm diverged from that of the others. In the worst scenario, it was 152.55 times faster than the ‘algorithm 1’ and 7.28 times faster than the ‘algorithm 3’.

**Fig. 9:**
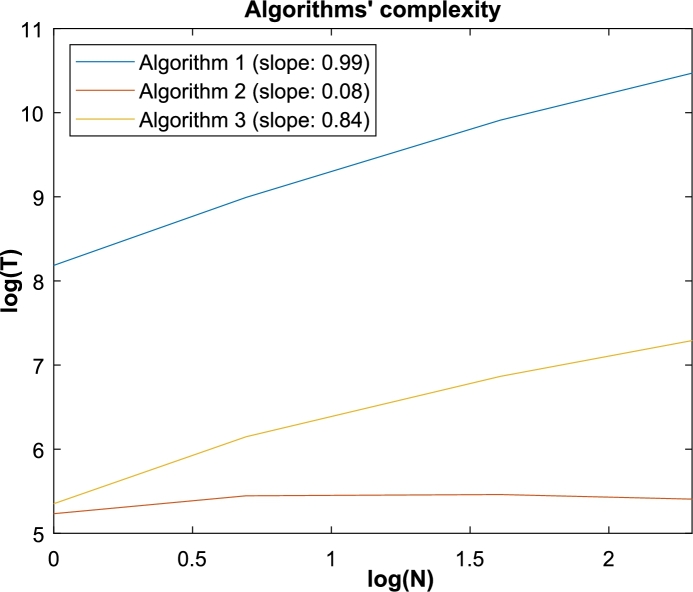
Complexity from the three algorithms based on the big-o annotation. T represents the time consumed to iterate the input of size N.

To compare the algorithm's complexity, the big-O notation was applied to describe how the runtime of the algorithms grows relative to the input size. Within this approach, the log from the average time consumed (‘T’) from each algorithm and from every input size (‘N’) is calculated, resulting in Fig. 9, where each algorithm has a slope ‘P’ in the complexity function $O(N^{P})$.

For the ‘algorithm 1’, the resulting complexity is near O(N), meaning that the runtime grows linearly in function of the input size. For the ‘algorithm 2’, the notation shows a nearly constant time (O(1)). In the ‘algorithm 3’, although sub-linear growth, it is still near to O(N), meaning that the required time still increases with the input size.

This result is also seen in the time usage, as the ‘algorithm 2’ is the only one that can constantly and significantly reduce the time when the input increases.

Therefore, in both the algorithms' analysis, in terms of time usage and in the big-O notation, ‘algorithm 2’ achieved the best results, showing that the combination of bit-wise communication with vector reshape operations is the most efficient.

### 3.4 Noise analysis

The first validation step is to calculate the input-referred noise from each channel of the PCB. This showcases how much noise may be generated from the PCB without considering external factors besides the power-line induction on the PCB itself. This test was made by using the short-input option from the CHxSET registers, acquiring a sample signal of at least 30 s for each channel with the PGA configured to 1, 6 and 12 to analyse the PGA effect on the noise, and sampling between 1, 2 and 4 kHz. The result is presented in Table 3.

**Table 3:** PCB input-referred noise in μV${}_{\text{rms}}$ with differential inputs short-circuited and resulting noise transmitted to GUI.

*f*_*s*_	Channel	Programmable Gain		
		1	6	12
1 kHz	CH1	3.1290	0.1624	0.1493
	CH2	3.0951	0.1383	0.1490
	CH3	3.1717	0.1645	0.0693
	CH4	3.0759	0.1364	0.0624
	CH5	3.0588	0.1237	0.0554
	CH6	3.3482	0.1440	0.0554

2 kHz	CH1	4.3066	0.2408	0.0883
	CH2	4.2789	0.1929	0.0936
	CH3	4.3864	0.1732	0.0886
	CH4	4.2355	0.1671	0.0736
	CH5	4.2514	0.1804	0.0672
	CH6	4.6464	0.1691	0.0792

4 kHz	CH1	6.4391	0.2745	0.1218
	CH2	6.5022	0.2679	0.1238
	CH3	6.5755	0.2714	0.1103
	CH4	6.4869	0.2581	0.1060
	CH5	6.4569	0.2538	0.0943
	CH6	6.6943	0.2736	0.0971

With the results shown in Table 3 it is concluded that increasing the PGA gain decreases the noise. In the same aspect, the slightest noises were measured with lower sampling frequencies. This shows an advantage in maintaining the acquisition with the highest gain and with the 1 kHz sampling unless otherwise required.

The higher sampling frequency behaviour is an effect of the delta-sigma architecture of the ADC. Since the ADC always samples the signal at 512 kHz and then averages multiple samples to achieve the configured data rate, when the required data rate increases (i.e., from 1 kHz to 2 or 4 kHz), the number of samples per average decreases, increasing the resulting noise [30].

Furthermore, the noise reduction when increasing the PGA is explained by the PGA bandwidth behaviour when varying the gain. Following the IC datasheet and the configuration proposed here, it can be established that the PGA bandwidth starts at 237 kHz when PGA is 1, goes to 64 kHz with PGA 6, and reaches its minimum bandwidth of 32 kHz with PGA 12. This showcases that the ADC is reducing the amount of noise being amplified.

With a maximum input-referred noise of 6.7 μV${}_{\text{rms}}$, it is proven that very small noise magnitudes are generated in the PCB and that the higher the configured PGA, the higher the signal-to-noise ratio. Furthermore, except for the configuration of 4 kHz sampling frequency with gain 1, all the other accomplish the required 5 μV${}_{\text{rms}}$ [27].

Considering the worst-case of 6.7 μV${}_{\text{rms}}$, with a 24-bit (*R*) ADC scale range from -2.4 ($V_{REF-}$) to 2.4 V ($V_{REF+}$), the resulting minimum effective number of bits (ENOB) regarding input-referred noise ($N_{ir}$) is estimated by the equation

(1) $$\text{ENOB}=24-\log_{2}\left(\frac{N_{ir}}{\frac{V_{REF+}-V_{REF-}}{2^{R}}}\right),$$

resulting in an ENOB between 19 and 20 bits in the worst scenario.

### 3.5 Data validation

The next validation step was to identify how trustworthy a signal acquired with the module, transmitted and stored using the interface is. Therefore, a known sine wave was generated using an AFG1022 Tektronix® function generator and connected to channel 1. The sampling frequency was kept at 1 kHz and with a 12-time PGA gain. The signal's amplitude and frequency were tested between 20 to 400 mVpp and 1 to 200 Hz. The voltage ranges from the function generator's minimum peak-to-peak voltage with minimum noise up to the ADC saturation level. As the voltage increases, the PGA makes the signal approaches the ADC saturation state (i.e., as ${\text{V}}_{\text{REF}}$ = 2.4 V is defined in CONFIG1, the ADS129x ADC voltage ranges from -2.4 V up to 2.4 V and the 400 mVpp sine varies from -200 to 200 mV, which multiplied by the PGA gain, reaches 2.4 V), limiting the maximum amplitude in 400 mVpp.

The acquired signal was transmitted to the interface, where at least 30 s were stored. Then, a digital signal was generated and compared with the resulting file downloaded from the interface. To align the acquired signal with the digitally synthesised, the original signal is shifted to have its maximum point on the first index, and the synthetical is generated as a cosine to start at maximum. Then, all the signals were limited to a 30 s length, and the signal normalised mean squared error (NMSE%) and Pearson correlation coefficient (PCC%) were calculated in reference to the synthetical signal, in addition to the original signal total harmonic distortion (THD [dBc]).

The NMSE% was calculated through the equation

(2) $$\text{NMSE}\text{\%}=\frac{\frac{1}{N}\sum_{i=1}^{N}{\left(A_{i}-B_{i}\right)}^{2}}{\text{max}{\left(A\right)}^{2}}\times 100\text{\%}$$

where *A* is the synthetic signal, *B* is the acquired signal, and *N* is the length of them in samples (for 30 s, equals 30 kS)

For the PCC% (*ρ*), the equation

(3) $$\rho (A,B)=\frac{\sum_{i=1}^{N}\left(\frac{A_{i}-\mu_{A}}{\sigma_{A}}\right)\times \left(\frac{B_{i}-\mu_{B}}{\sigma_{B}}\right)}{N-1}\times 100\text{\%}$$

is used, where *A* and *B* represents both signals, *μ* is the mean and *σ* is the standard deviation. The closer *ρ* is from 100%, the more correlated the signals are.

For the THD, the signal is converted to the frequency domain through FFT (fast Fourier transform). Then, the RMS voltage of the fundamental frequency's harmonics ($V_{k}$), up to the last before the Nyquist frequency, is measured and compared with the carrier RMS voltage ($V_{1}$). This is explained by the equation

(4) $$\text{THD}=10\cdot \log_{10}\left(\frac{\sum_{k=2}^{\left\lfloor \frac{f_{s}}{2⁎f_{1}}\right\rfloor }V_{k}^{2}}{V_{1}^{2}}\right)\text{[dBc]}.$$

The results are shown in Table 4, where the behaviour between higher frequency causing visually higher noise is noticeable. As frequency increases, it reaches values closer to the Nyquist limit ($f_{s}/2$), and since no interpolation is made, the result diverges visually from the shape of a sinusoidal wave; however, since the PCC% is kept high and the THD increases, the NMSE% is demonstrated as a visual fault.

**Table 4:** Calculated normalised mean square error (NMSE%) and Pearson correlation coefficient (PCC%) from function generator sinusoidal wave converted to digital, transmitted and stored, compared with a digitally synthesised wave, and, total harmonic distortion (THD [dBc]) from the original stored signal.

Frequency	Measure	Amplitude				
		20 mVpp	100 mVpp	150 mVpp	200 mVpp	400 mVpp
1 Hz	NMSE%	0.00627	0.00081	0.0002	0.00005	0.0001
	PCC%	100.0	100.0	100.0	100.0	100.0
	THD	-56.89	-60.74	-64.29	-70.17	-63.59

10 Hz	NMSE%	0.01123	0.00038	0.00452	0.00967	0.00297
	PCC%	99.96	100.0	99.98	99.96	99.99
	THD	-56.03	-62.2	-66.1	-68.31	-65.22

100 Hz	NMSE%	0.46047	0.32828	0.43976	0.07903	0.48533
	PCC%	98.15	98.72	98.26	99.78	98.08
	THD	-60.03	-64.8	-70.22	-73.46	-68.66

200 Hz	NMSE%	0.59264	0.98633	1.07644	1.88901	0.93097
	PCC%	98.95	97.15	96.71	92.76	97.44
	THD	-64.32	-71.3	-77.08	-76.15	-76.52

Furthermore, the higher noise on the 20 mVpp samples may be explained by the function generator's higher noise in creating low voltage sine waves, as its error is intrinsically related to the signal-to-noise ratio, becoming more evident as the signal amplitude decreases.

Finally, to test the ADC with frequency-domain complex signals, a multiple-sinc wave was generated, as it resembles a cardiac pulse-like signal. The signal was generated in the function generator, with maximum amplitude fixed at 150 mV and minimum at 0 V. The number of side lobes was generated based on the generator aspect. The fundamental frequency $f_{0}$ was tested at 0.6 Hz (lower than a heart's minimum expected beats per minute (BPM)) and at 5 Hz (higher than the maximum expected BPM). Every sinc-wave had $40⁎f_{0}⁎\pi$ side lobes but were truncated to force one sinc-peak every $1/f_{0}$. Both sinc-waves were acquired with the module, using channel 6, 12x gain and 1 kHz sampling rate. The acquired and synthesised signals were compared with the same NMSE% and PCC% in the time and frequency domains, normalising the FFT to calculate the NMSE%. The results are shown in Table 5.

**Table 5:** Calculated normalised mean square error (NMSE%) and Pearson correlation coefficient (PCC%) from time-domain and frequency-domain of multiple-sinc wave acquired with the module compared with a digitally synthesised.

*f*_0_	Measure	Domain	
		Time	Frequency
0.6 mHz	MSE%	0.149396	0.000016
	PCC	96.00	99.68

5 Hz	MSE%	0.038759	0.000006
	PCC	99.31	99.90

Analysing the results from Table 5, with Pearson correlation coefficients exceeding 96% in both the time and frequency domains and normalised mean square errors (NMSE%) below 0.15% in time and even lower in frequency, the system demonstrates strong accuracy and minimal distortion. The slightly better performance in the frequency domain suggests that the system is particularly effective at preserving spectral content. This proves that the system is highly capable and provides reliable signal acquisition with minimal noise and distortion.

## 4 Discussion and conclusion

This article proposes a novel biosignals acquisition module for non-specific research purposes. The main novelty of the module is its highly configurable characteristic, allowing the acquisition of ECG and sEMG in parallel, with up to 8 different channels.

The research resulted in a portable module that is compatible with medical-grade cables and other commonly used 3-pole jack cables for better user experience. This is an improvement to commercial devices, where company-specific cables are required (e.g., BITalino) [24], soldered cable [26], or built-in electrodes (e.g., KardiaMobile6L) [25].

The system also brings a high-resolution ADC with configurable sampling frequency while maintaining wireless data transmission. While the presented system wireless acquires a 24-bit signal of 8 channels, commercial devices focus on resolutions smaller than 16 bits, with fixed sampling frequencies close to the Nyquist limit. To achieve 24-bit resolution, devices use wired data transmission [25]. This is achieved by employing a bit-wise transmission approach; the packet was reduced by 83.92% if compared to JSON formatting. In the same topic, the decoding algorithm was also optimised to reduce the required time between iterations, being up to 7.28 times faster than using JSON and using a maximum of 0.19% from the time available between iterations for the decoding process.

During tests, the module achieved more than 22 hours of acquisition and wireless transmission when powered by an 18650 3350 mAh Li-Ion battery. The input-referred noise from the module's channels achieved a maximum value of 6.7 μV${}_{\text{rms}}$. When comparing a sine wave acquired using the module with a digitally synthesised one, it achieved correlations higher than 99.99%.

The system also achieved a maximum total harmonic distortion of -53 dBc in the worst case an average of -66.8 dBc and a minimum of -77.08 dBc (best case), while the BITalino (r)evolution achieved a minimum of -59.80 dBc [24], and other research achieved a minimum of -45.53 dBc [31].

Considering the worst-case, the system maintains a strong signal correlation (PCC% > 92%) and low harmonic distortion (THD < -64 dBc), higher than the requirements for biosignal acquisition module [32], [33], confirming the effectiveness of the acquisition module for low-to-mid frequency analogue waveforms, which comprises the electrocardiogram [34] and electromyogram [35] bandwidth of interest. This concludes that the system can reliably acquire, transmit and store harmonic signals.

The analysis and comparison of biosignals can be highly subjective, making it difficult to standardise results compared to a commercial medical-grade acquisition device. Other means to validate the acquisition were used, such as analysing the noise and the transmission trustworthiness, but the comparison with signals acquired with different devices is still a limitation.

Furthermore, inside the device validation regarding its noise, when the signal acquired from the function generator is compared with an algorithmically generated wave, the real efficiency can be decreased based on generator noise instead of an acquisition problem, falsely reducing the system's credibility. In most cases, this was not a problem since the system accuracy was kept high with different parameters, which was a limitation with a very low voltage signal.

However, compared with the literature, the system requires improvement in different aspects. As further works, it is proposed to enhance the control interface with more simultaneous graphs and feature extraction functionalities, increasing the system's usability. Other wireless communication protocols will be tested and implemented to validate against classic Bluetooth® regarding energy consumption and data volume. Further on energy consumption, the flexibility will be implemented in packet structure to allow for low-power operation of the ADC, reducing consumption and resolution. It is also intended to apply for the required ethical approval to acquire signals from volunteers and create a diverse dataset to test the proposed feature extraction functionalities and mitigate bias during validation.

## Ethics statement

The signals presented during this work and used for validation were acquired from the main author, Luiz E. Luiz. No other human subject was used during the experimentation. This work did not require ethical clearance.

## Declaration of generative AI and AI-assisted technologies in the writing process

During the preparation of this work, the authors used Grammarly to improve language and readability. After using this tool, the authors reviewed and edited the content as needed and take full responsibility for the content of the publication.

## CRediT authorship contribution statement

**Luiz E. Luiz:** Writing – original draft, Visualization, Software, Methodology, Investigation, Formal analysis, Data curation, Conceptualization. **Salviano Soares:** Writing – review & editing, Supervision. **Antonio Valente:** Writing – review & editing. **João Barroso:** Writing – review & editing, Supervision. **Paulo Leitão:** Writing – review & editing, Supervision, Resources, Funding acquisition. **João P. Teixeira:** Writing – review & editing, Validation, Supervision, Resources, Project administration, Funding acquisition, Formal analysis.

## Declaration of Competing Interest

The authors declare that they have no known competing financial interests or personal relationships that could have appeared to influence the work reported in this paper.
